# Part-solid tumours: at the border of 2 worlds

**DOI:** 10.1093/icvts/ivab305

**Published:** 2021-10-28

**Authors:** Paul E Van Schil, Lawek Berzenji

**Affiliations:** Department of Thoracic and Vascular Surgery, Antwerp University Hospital, Edegem, Belgium; Department of Thoracic and Vascular Surgery, Antwerp University Hospital, Edegem, Belgium

**Keywords:** Non-small cell lung cancer, Ground-glass opacity, Part-solid tumour, Lobectomy, Sub-lobar resection, Surgery

In the eighth edition of the Tumour—Node—Metastasis (TNM) classification a new T descriptor was introduced with only the invasive part taken into account to determine the size of part-solid nodules [1, 2]. In most instances, this corresponds to the solid part of the nodule on computed tomographic (CT) scanning. Therefore, a distinction is made between pure, 100% ground-glass opacities (GGO) without any solid component, part-solid lesions with a GGO part between 1 and 99%, and 100% solid lesions without any GGO component. Sub-solid lesions comprise GGO and part-solid lesions together. Equally, in the new adenocarcinoma classification, a division is made between adenocarcinoma *in situ*, minimally invasive adenocarcinoma and invasive adenocarcinoma [3]. Management of these lesions remains a hotly debated topic focussing on the indications for surgical resection and the role of sub-lobar resection comprising wedge excision and anatomical segmentectomy [4, 5].

In the present manuscript, the authors evaluate a large series of 1061 patients who underwent lung resection and for whom thin-section CT images were available to determine the clinical T factor [6]. Patients were treated in 2 dedicated Japanese centres. Postoperative 5-year survival rates of patients with pure ground-glass tumours, part-solid lesions and solid tumours were 97.6%, 89.0% and 76.3%, respectively. For these survival calculations, the whole tumour size was taken into account. Considering all patients with part-solid lesions and solid tumours, multivariate analysis showed that age <70 years, female sex, interventions comprising lobectomy or more, size of solid component and part-solid tumours were independent predictors of better survival. The authors conclude that patients with part-solid tumours show a significantly better survival than those with solid tumours and that this specific finding should be considered in the upcoming ninth edition of the TNM classification due in 2024.

The present study providing an in-depth analysis of the clinical T descriptor in the eighth TNM classification for part-solid lesions is clinically relevant. The authors show that part-solid tumours have an intermediate prognosis with an overall survival rate in between pure GGO lesions and 100% solid tumours.

Although a large number of patients are included and a thorough analysis is performed, it is a retrospective study with its inherent limitations resulting in a lower grade of evidence for the final recommendations. The study period extends over a 10-year period during which imaging techniques were significantly improved. Moreover, patients were selected from 2 hospitals with different CT scanners with different slice thicknesses taken into account (1 vs 2 mm). No stratification per centre is presented. Quite surprisingly, there are no thoracic radiologists as co-authors. Although Japanese thoracic surgeons have extensive experience in evaluating CT images, this may introduce another bias as no interpretation of CT examinations was performed by independent thoracic radiologists.

As is often the case in retrospective studies, quite a lot of patients had to be excluded. Of the initial total of 1538 patients, detailed CT images were available in only 1061 cases; another 196 patients were excluded due to insufficient follow-up, resulting in a total of 673 patients who were excluded (43.7%).

Regarding sub-lobar resections, no specific distinction could be made between anatomical segmentectomies and (wide) wedge excisions which may have a different prognosis, especially for invasive tumours >2 cm. Equally, no correlation between survival and specific subcategories of adenocarcinomas could be provided. No information is given on the number of patients with adenocarcinoma *in situ*, minimally invasive adenocarcinoma, lepidic predominant, acinar, papillary, micropapillary or solid adenocarcinoma which have a different prognosis. Specifically, micropapillary and solid adenocarcinoma have a worse prognosis than the other subtypes. Were there any cases with spread through air spaces, which has a poorer prognosis [7]?

Regarding the survival data, only overall survival data are provided and causes of death are not listed. Disease-free survival is more relevant for clinical T1 tumours as particularly patients with smaller tumours may die from causes unrelated to their lung cancer.

Regional variation should also be taken into account as sub-solid lesions seem to be particularly prevalent in Asian countries. So, data from Asia cannot be extrapolated to other countries or continents. For this reason, centres with a large experience in treating these tumours are encouraged to participate in the prospective database of the International Association for the Study of Lung Cancer (IASLC), currently also including molecular data [8]. These can be used for refining the T descriptor in the TNM classification. For the time being, it is recommended to register the invasive size as well as the total size of part-solid lesions [2]. Pathological findings have to be compared with CT images to provide a comparison between the clinical (imaging) and pathological T descriptor and to determine which is the most important prognostic factor in different parts of the world [9]. It should also be pointed out that the ‘solid’ part on CT sections does not necessarily correspond to ‘solid’ adenocarcinoma which is a distinct pathological entity.

In conclusion, this paper provides some additional evidence that part-solid lesions have combined benign and malignant features resulting in an overall survival rate between pure GGO lesions and 100% solid lesions. Due to its retrospective design, not all relevant prognostic factors could be studied letting the authors finally to conclude that ‘A well-designed prospective study with a large number of patients is warranted to clarify the prognostic significance of part-solid tumours’ [6].
